# Understanding the apprehension and concern haunting patients before a total knee arthroplasty

**DOI:** 10.1186/s42836-021-00069-5

**Published:** 2021-03-29

**Authors:** Sanjay Bhalchandra Londhe, Ravi Vinod Shah, Meghana Patwardhan, Amit Pankaj Doshi, Shubhankar Sanjay Londhe, Kavita Subhedar

**Affiliations:** 1 Orthopedic surgeon, Holy Spirit Hospital, Mahakali Caves Road, Andheri east, Mumbai, Maharashtra 400093 India; 2 Orthopedic Surgeon, Criticare Superspeciality Hospital, Andheri East, Mumbai, Maharashtra India; 3 Interventional Pain & Palliative Care Physician, Criticare Superspeciality Hospital, Andheri East, Mumbai, Maharashtra India; 4 Meril Life Sciences, Mumbai, India; 5 Dr Vishwanath Karad MIT World Peace University, Pune, India; 6 Criticare Superspeciality Hospital, Andheri East, Mumbai, India

**Keywords:** Total knee arthroplasty, Fear and apprehension, Postoperative pain, Patient counseling

## Abstract

**Purpose:**

The aim of this prospective study was to understand the fear and apprehension factors that play on patient’s mind before total knee arthroplasty.

**Methods:**

This retrospective cohort study included 500 consecutive patients (375 females and 125 males) who were scheduled to undergo total knee arthroplasty the next day. The patients were asked to list the most important fear in their mind regarding the operation in descending order of importance. They were given a questionnaire form which contained several capture points, including age, gender, educational background, occupation, and provision of help at home. Preoperative pain was measured by using the visual analog score, and its influence on the patients’ fear and apprehension factors was also measured.

**Results:**

In this study, 58% of patients (50 males, 40%; 240 females, 64%) were fearful of the pain that they would experience after surgery and during the postoperative physiotherapy. The female patients showed more fear of pain than their male counterparts (*P* < 0.05). 18% of the patients (40 males, 32%; 50 females, 13%) listed whether they will be able to walk and perform activities of daily living after surgery as the most important fear. The male patients had more fear of returning to normal walking (*P* < 0.05). 20% of the patients (30 males, 24%; 70 females, 19%) were fearful about getting adequate home help after discharge from hospital (*P* > 0.05). 4% of patients were concerned about withstanding such a major operation. There was no difference between male and female patients (*P* > 0.05).

**Conclusion:**

The majority of the patients experience apprehension of pain in the perioperative period of TKA. Preoperative counseling benefits pain management by alleviating the patient’s concerns about the fear of postoperative pain and apprehension of returning to normal walking.

**Supplementary Information:**

The online version contains supplementary material available at 10.1186/s42836-021-00069-5.

## Introduction

A large population-based prevalence surveys showed that, with increased life expectancy and obesity, the incidences of the degenerative knee diseases stood at around 10% [1]. Total knee arthroplasty (TKA) is one of the most successful surgeries in modern-day orthopedics, and the number of surgeries performed is on the rise by the year worldwide [2].

Multiple studies have shown that the TKA could markedly improve patients’ knee pain, physical function, and health-related quality of life [1, 2]. In the developing countries, however, a sizable number of patients refuse to undergo TKA due to certain factors, including perioperative fear and apprehension of pain, other anxiety, age, gender, area of residence, education level, occupation, expectation, cost, *etc*. Moreover, in some cases, the decision may be made by patients’ family. Understanding patient’s decision making has potential benefits to improve preoperative counselling, patient satisfaction and financial constraints.

The aim of this prospective study was to understand the fear and apprehension factors that play on patient’s mind before TKA.

## Materials and methods

The institutional review boards of the participating hospitals approved the study. Informed consent was obtained from each patient.

A total of 500 consecutive patients (375 females, 75%; 125 males, 25%) were recruited for this study. Our inclusion criterion was a patient who underwent a primary TKA. We excluded the patients who underwent a revision arthroplasty because a higher pain tolerance was likely. The mean age of female patients was 73 years (range, 66 to 82 years). The mean age of male patients was 74 years (range, 70 to 85 years).

Before surgery, the patients who were planned to undergo the TKA were asked to list the foremost important fear in their mind regarding the operation in the descending order of importance. They were given a questionnaire form including several capture points like age, gender, educational background, occupation, and help they receive (Annexure 1). The severity of fear and apprehension was scored on a 1-to-3 scale (1, mild fear; 2, moderate fear; 3, major or severe fear). Their responses were recorded as numerical values, ranging from 4 to 12 (Table 1). An independent observer distributed the questionnaire and the patients filled out the questionnaire and handed it over to the observer. The results were interpreted by another independent observer. Preoperative knee pain was measured by using the 10-point visual analog score, and its influence on the patients’ fear and apprehension was also measured. The patients who underwent unilateral and bilateral TKAs were also compared to find whether there was a difference between those patients in their fear and apprehension. The patients who scored 7 or above were given extra counseling during the perioperative period. Six months after TKA, we asked the patients again to confirm whether they experienced preoperative apprehension and fear. This investigation was done through a telephon interview by the same independent observer.

**Table 1:** Numerical score of fear/apprehension factors

Fear/ Apprehension	Severity of Fear/Apprehension	Grade/Score	Score
**Fear of Pain**	Mild	I	**Maximum sub-score = 3**
	Moderate	II	
	Severe	III	
**Fear of receiving help at home**	Mild	I	**Maximum sub-score = 3**
	Moderate	II	
	Severe	III	
**Fear of returning to activities of daily living**	Mild	I	**Maximum sub-score = 3**
	Moderate	II	
	Severe	III	
**Fear of withstanding surgery**	Mild	I	**Maximum sub-score = 3**
	Moderate	II	
	Severe	III	
**Total sum score**			**Total maximum score = 12**

The chi-square test was employed for statistical analysis. The degree of freedom was 1, and a *P* value < 0.05 was considered statistically significant.

## Results

**Fig. 1:**
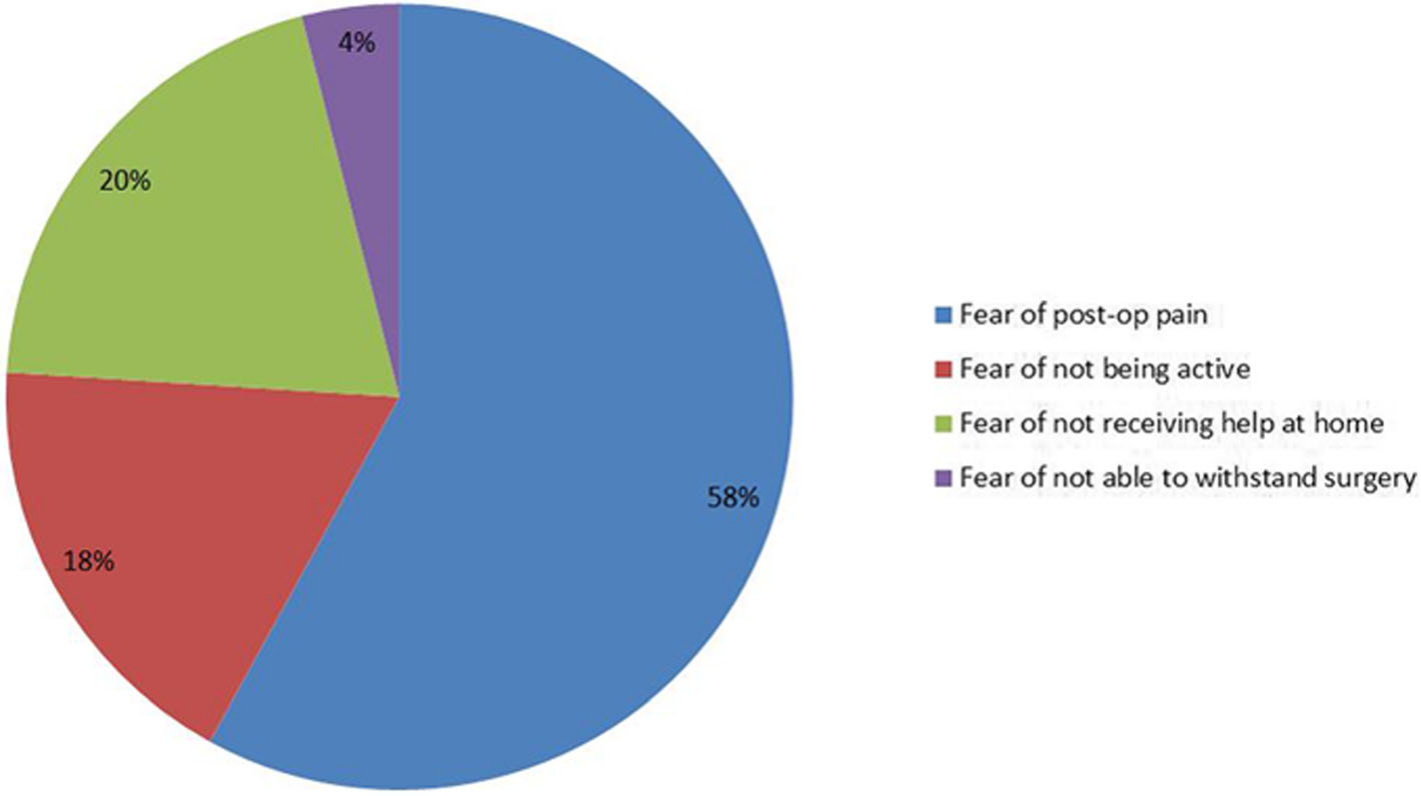
Pie chart showing percentage distribution of different fear/ apprehension factors

**Fig. 2:**
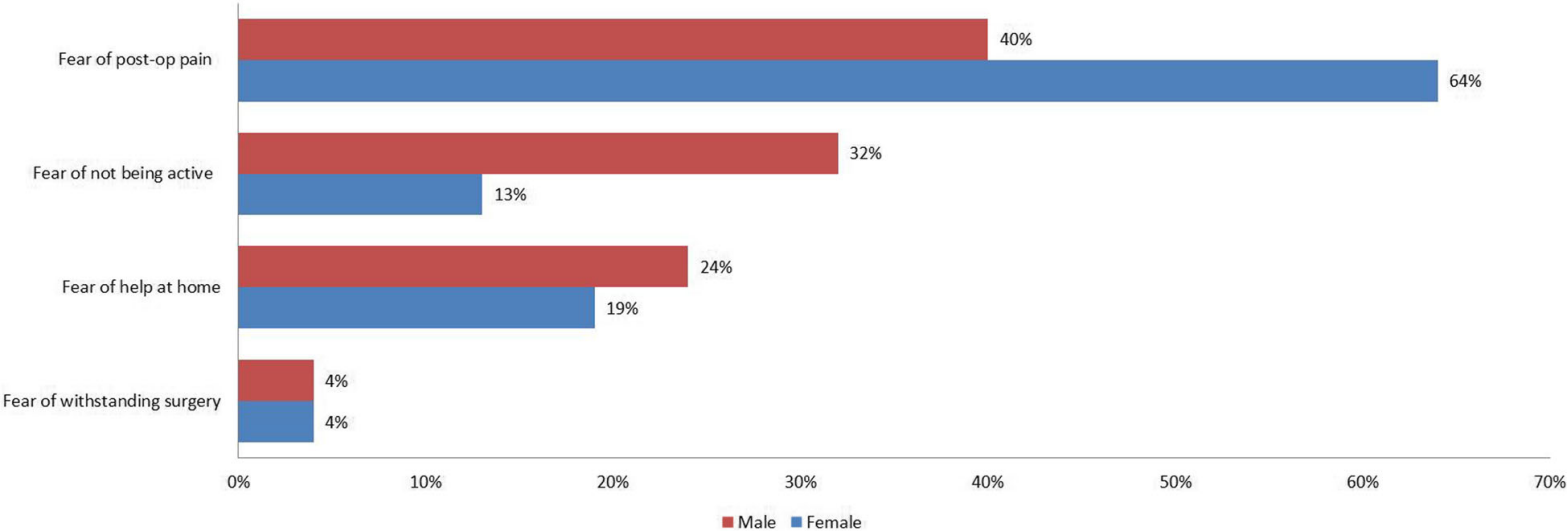
Bar diagram showing distribution of fear/ apprehension factors in different gender groups

All the 500 patients willingly participated in the study. Among them, 58% (50 males and 240 females) reported apprehension of the pain related to the TKA and physiotherapy; 18% of the patients (40 males and 50 females) listed whether they would be able to walk and do activities of daily living (ADL) after TKA, which was the most important fear; 20% (30 males and 70 females) were fearful of whether they would get adequate home help once they get discharged from hospital and 4% (5 males and 15 females) concerned whether they would be able to withstand such a major operation (Figs. 1 and 2). Regarding gender, 40% of the male patients (*n* = 50) and 64% of the female patients (*n* = 240) listed fear of pain as the most important apprehension. Female patients had significantly more fear of pain than their male counterparts (chi-square = 4.4335; degree of freedom = 1; *P* = 0.0352); 32% of the male patients (*n* = 40) and 13% of the female patients (*n* = 50) reported strong apprehension whether they would return to the normal walking and ADL. The male patients suffered from more fear on whether they would be able to return to the normal walking and ADL than female patients (chi-square = 4.4263; degree of freedom = 1; *P* = 0.0354); 24% of the male patients (*n* = 30) and 19% of the female patients (*n* = 70) primarily concerned whether they would be able to receive a domestic assistance at home after their discharge from the hospital (chi-square = 0.3333; degree of freedom = 1; *P* = 0.5637); 4% of both male (*n* = 5) and female (*n* = 15) patients listed the fear of withstanding the major surgery as the most important apprehension (*P* = 1.000) (Table 2). Patients whose preoperative visual analog score > 7 reported more apprehension about returning to normal ADL than the fear of pain (Table 3). There was no statistically significant difference regarding the fear of pain, returning to normal ADL, or fear of withstanding the major surgery between patients who underwent unilateral TKAs and those receiving bilateral TKAs. Fear of not receiving help at home was more severe in the patients who underwent bilateral TKA (Table 4). Six months after TKA, the same independent observer asked the patients to report whether their preoperative apprehension and fear were unfounded. This investigation was done through a telephon interview. In this study, 95% of the patients (*n* = 475) reported that their preoperative fear and apprehension were exaggerated; 5% of patients (*n* = 25) had genuine difficulty regarding the arrangement of help at home after discharge from hospital.

**Table 2:** Gender-wise breakup of fear / apprehension factors with *p* values

	Male	Female	***P*** value
**Total number of patients**	125	375	
**Fear of pain**	50	240	0.03524
**Fear of return to normal activities / ADL**	40	50	0.03539
**Apprehension about receiving domestic help**	30	70	0.5637
**Fear of withstanding major surgery (TKA)**	5	15	1.000

**Table 3:** Comparison in terms of preoperative VAS score with *P* values

	Patient’s with VAS ≤ 7	Patient’s with VAS > 7	***P*** value
**Total number of patients**	160	340	
**Fear of pain**	103 (64.37%)	187 (55%)	0.0479
**Fear of return to normal activities / ADL**	18(11.25%)	72 (21.17%)	0.0071
**Apprehension about receiving domestic help**	33 (20.26%)	67 (19.70%)	0.8106
**Fear of withstanding major surgery (TKA)**	6 (3.75%)	14 (4.12%)	0.8453

**Table 4:** Comparison between unilateral and bilateral TKA with *p* values

	Unilateral TKA	Bilateral TKA	*P* value
**Total number of patients**	420	80	
**Fear of pain**	245 (58.33%)	45 (56.25%)	0.7300
**Fear of return to normal activities / ADL**	81 (19.28%)	9 (11.25%)	0.0869
**Apprehension about receiving domestic help**	77 (18.33%)	23 (28.75%)	0.0329
**Fear of withstanding major surgery (TKA)**	17 (4.04%)	3 (3.75%)	0.9035

## Discussion

This study highlights that apprehension and fear are important for the patients who are scheduled to undergo TKA. Majority of the patients in our cohort were concerned about the postoperative pain, and the female patients showed more fear about postoperative pain than male patients. The male patients had more fear than female patients regarding returning to the normal walking and ADL. The female and male patients showed nearly equal fear about the help they receive at home during the postoperative period and the fear of withstanding the major surgery.

Said *et al*. [3] showed that the African-American patients were less likely to consider TKA than the white patients, because the former’s perception of hospital course, pain, and functional recovery might be different. Vina *et al*. [4] found that social support affected patients’ preference for TKA in the white patients rather than in the African-American patients. Liang *et al*. [5] demonstrated that the TKA is a cost-effective surgery for the advanced osteoarthritis of the knee joint, especially in aging populations. In developing countries, however, 65% of patients refuse to receive TKA due to the certain factors, such as the high total cost, the fear of complications, *etc*. [6] Zhao *et al*. [7] also suggested the patient factors contributed to the decision to delay TKA. The fear of pain is the number one factor in the initial postoperative weeks, and next comes the interference with work. Other factors that affect the decision-making include gender and income, and educational background does not significantly affect the decision-making. Sharifa *et al*. [8] conducted a systematic research of the databases like MEDLINE, PubMed, *etc*. They found that the preoperative anxiety is the most important predictor of poor outcomes (in terms of higher levels of knee disability and poor WOMAC score) and poor quality of life one year after surgery [9–12].

Postoperative pain can be alleviated by epidural analgesia, regional blocks (femoral and adductor canal blocks), neuropathic pain modulators (pregabalin), *etc*. Ideally, postoperative pain should be managed by a team composed of orthopaedic surgeon, anesthetist, pain consultant, and rehabilitation nurse. Hence, preoperative counseling of patients is helpful for the multimodal management of pain. The patients are shown that various perioperative strategies are available for improving their quality of life, desirably through the audio-visual demonstrations. We give the patients our contact numbers so that they can reach us to report their improvement. In order to boost self-confidence of the patients to be operated, we arrange them to have an additional preoperative interactive session with the patients who have already undergone TKA. The contact numbers are included in the patient information booklets.

Our study has limitations. Some factors, such as the cost of surgery, social network, and patients’ own psychological framework, also influence the patients’ decision to undergo TKA, but those factors were not included. Further studies are needed to ascertain the number of patients who refuse to undergo TKA due to the fear and apprehension, and the number of patients who have already overcome the fear and apprehension after consulting doctors about the surgical procedure and postoperative management.

## Conclusion

Our study demonstrated that most of the patients experienced apprehension of pain in the perioperative period of TKA. Preoperative counseling benefits pain management by alleviating the patients’ fear about postoperative pain and apprehension of returning to normal walking.

## Supplementary Information

**Additional file 1.**

## Data Availability

Not Applicable.

## Abbreviations

TKA: Total Knee Arthroplasty
ADL: Activities of Daily Living
