# Supplementary material for: Understanding the apprehension and concern haunting patients before a total knee arthroplasty
Source: Arthroplasty. 2021 Mar 29;3:14. doi: 10.1186/s42836-021-00069-5 (PMC8796401; doi:10.1186/s42836-021-00069-5)
Supplement: Supplementary file 1 — Additional file 1. [file 42836_2021_69_MOESM1_ESM.doc]

**PATIENT SELF ADRESSED QUESTIONAIRE**

**Name**

**Age**

**Gender**

**Occupation**

**Qualification**

**Address**

**Contact information**

**Unilateral/ Bilateral TKR**

**Pre operative pain as measured by VAS Score on a scale of 0-10 (0=no pain to 10=worst pain)**

**Fear / Apprehension factors in decreasing order of importance**

**1. Pain after the operation**

**2. Whether ready to be able to withstand the surgery**

**3. What about help at home after discharge from the hospital**

**4. Return to activities of daily living (AOL) and normal walking**
